# Nosocomial infection update.

**DOI:** 10.3201/eid0403.980320

**Published:** 1998

**Authors:** R A Weinstein

**Affiliations:** Cook County Hospital, Division of Infectious Diseases, Chicago, IL 60612, USA. rweinste@rush.edu

## Abstract

Historically, staphylococci, pseudomonads, and Escherichia coli have been the nosocomial infection troika; nosocomial pneumonia, surgical wound infections, and vascular access-related bacteremia have caused the most illness and death in hospitalized patients; and intensive care units have been the epicenters of antibiotic resistance. Acquired antimicrobial resistance is the major problem, and vancomycin-resistant Staphylococcus aureus is the pathogen of greatest concern. The shift to outpatient care is leaving the most vulnerable patients in hospitals. Aging of our population and increasingly aggressive medical and surgical interventions, including implanted foreign bodies, organ transplantations, and xenotransplantation, create a cohort of particularly susceptible persons. Renovation of aging hospitals increases risk of airborne fungal and other infections. To prevent and control these emerging nosocomial infections, we need to increase national surveillance, "risk adjust" infection rates so that interhospital comparisons are valid, develop more noninvasive infection-resistant devices, and work with health-care workers on better implementation of existing control measures such as hand washing.


### Nosocomial Infection Update

**Robert A. Weinstein**
Cook County Hospital & Rush Medical College, Chicago, Illinois, USA

Historically, staphylococci, pseudomonads, and *Escherichia coli* have been the nosocomial infection troika; nosocomial pneumonia, surgical wound infections, and vascular access–related bacteremia have caused the most illness and death in hospitalized patients; and intensive care units have been the epicenters of antibiotic resistance. Acquired antimicrobial resistance is the major problem, and vancomycin-resistant *Staphylococcus aureus* is the pathogen of greatest concern. The shift to outpatient care is leaving the most vulnerable patients in hospitals. Aging of our population and increasingly aggressive medical and surgical interventions, including implanted foreign bodies, organ transplantations, and xenotransplantation, create a cohort of particularly susceptible persons. Renovation of aging hospitals increases risk of airborne fungal and other infections. To prevent and control these emerging nosocomial infections, we need to increase national surveillance, "risk adjust" infection rates so that interhospital comparisons are valid, develop more noninvasive infection-resistant devices, and work with health-care workers on better implementation of existing control measures such as hand washing.

As we enter the next millennium of infection control, we stand on the shoulders of giants—Jenner, Semmelweis, Nightingale, Oliver Wendell Holmes, and my own personal favorite, Thomas Crapper, the father of indoor plumbing. Modern infection control is grounded in the work of Ignaz Semmelweis, who in the 1840s demonstrated the importance of hand hygiene for controlling transmission of infection in hospitals. However, infection control efforts were spotty for almost a century. In 1976, the Joint Commission on Accreditation of Healthcare Organizations published accreditation standards for infection control, creating the impetus and need for hospitals to provide administrative and financial support for infection control programs. In 1985, the Centers for Disease Control and Prevention's (CDC's) Study on the Efficacy of Nosocomial Infection Control reported that hospitals with four key infection control components—an effective hospital epidemiologist, one infection control practitioner for every 250 beds, active surveillance mechanisms, and ongoing control efforts—reduced nosocomial infection rates by approximately one third (1).

Address for correspondence: Robert A. Weinstein, Cook County Hospital, Division of Infectious Diseases, 129 Durand, 1835 W. Harrison St., Chicago, IL 60612, USA; fax: 312-572-3523; e-mail: rweinste@rush.edu.

Over the past 25 years, CDC's National Nosocomial Infections Surveillance (NNIS) system has received monthly reports of nosocomial infections from a nonrandom sample of United States hospitals; more than 270 institutions report. The nosocomial infection rate has remained remarkably stable (approximately five to six hospital-acquired infections per 100 admissions); however, because of progressively shorter inpatient stays over the last 20 years, the rate of nosocomial infections per 1,000 patient days has actually increased 36%, from 7.2 in 1975 to 9.8 in 1995 (Table 1). It is estimated that in 1995, nosocomial infections cost $4.5 billion and contributed to more than 88,000 deaths—one death every 6 minutes.

Table 1. Nosocomial infections, United States (2,3)

| Year | Admissions ($x10^6$) | Patient days[a] ($x10^6$) | Length of stay (days) | Nosocomial infection ($x10^6$) | Nosocomial infections (/1000 patient days) |
|---|---|---|---|---|---|
| 1975 | 38 | 299 | 7.9 | 2.1 | 7.2 |
| 1995 | 36 | 190 | 5.3 | 1.9 | 9.8 |

[a]Patient days = total inpatient days.

### Which Nosocomial Infections Are Emerging?

We have witnessed a cyclical parade of pathogens in hospitals. In Semmelweis's era, group A streptococci created most nosocomial problems. For the next 50 to 60 years, gram-positive cocci, particularly streptococci and *Staphylococcus aureus*, were the hospital pathogens of major concern. These problems culminated in the pandemic of 1940 to 1950, when *S. aureus* phage type 94/96 caused major nosocomial problems. In the 1970s, gram-negative bacilli, particularly *Pseudomonas aeruginosa* and *Enterobacteriaceae*, became synonymous with nosocomial infection. By the late 1980s and early 1990s, several different classes of antimicrobial drugs effective against gram-negative bacilli provided a brief respite. During this time, methicillin-resistant *S. aureus* (MRSA) and vancomycin-resistant enterococci (VRE) emerged, signaling the return of the "blue bugs." In 1990 to 1996, the three most common gram-positive pathogens—*S. aureus*, coagulase-negative staphylococci, and enterococci—accounted for 34% of nosocomial infections, and the four most common gram-negative pathogens—*Escherichia coli*, *P. aeruginosa*, *Enterobacter* spp., and *Klebsiella pneumoniae*—accounted for 32% (3).

Bloodstream infections and pneumonias have increased in frequency from 1975 to 1996 (Table 2). However, tracking nosocomial infections by site has become difficult in the last few years because of shorter inpatient stays. For example, the average postoperative stay, now approximately 5 days, is usually shorter than the 5- to 7-day incubation period for *S. aureus* surgical wound infections.

Acquired antimicrobial resistance is the major anticipated problem in hospitals. VRE and MRSA are the major gram-positive pathogens of concern (5,6). *P. aeruginosa*, *Klebsiella*, and *Enterobacter* that harbor chromosomal or plasmid-mediated beta-lactamase enzymes are the major resistant gram-negative pathogens.

Table 2. Sites of nosocomial infections (2,4)

| Year | Urinary tract (%) | Surgical wound (%) | Lower respiratory tract (%) | Bloodstream (%) | Other (%) |
|---|---|---|---|---|---|
| 1975 | 42 | 24 | 10 | 5 | 19 |
| 1990–96 | 34 | 17 | 13 | 14 | 21 |

The contribution of antibiotic resistance to excessive death rates in hospitals is difficult to evaluate, often depending on whether studies are population-based or case-control, but evidence is mounting that antimicrobial resistance contributes to nosocomial deaths.

While bacterial resistance is clearly the major threat, viral and fungal resistance could become important because of the small number of therapeutic options for these pathogens. Herpes viruses with acquired resistance to acyclovir and ganciclovir have emerged as problems, particularly in HIV-infected patients. Pathogens with intrinsic resistance often have lower pathogenicity and have disproportionately affected immunocompromised patients. For example, *Candida* spp. with intrinsic resistance to azole antifungal agents (e.g., *C. krusei*) and to amphotericin B (e.g., *C. lusitaniae*) have emerged as problem pathogens in oncology units.

While we are facing the era of opportunists, including fungi, viruses, and parasites in immunocompromised patients, the one we fear most is the postantibiotic era. The first nosocomial inkling is MRSA with reduced susceptibility to vancomycin (7). Beyond the postantibiotic era lies the era of xenogenic infections as organs, transplanted from nonhuman primates, bring with them a variety of potential zoonotic pathogens. Nevertheless, traditional respiratory pathogens may yet prove to be our greatest challenge; for example, a major shift in strain type (8) could result in devastating pandemic community and nosocomial influenza A outbreaks.

### Who Is Affected by Emerging Nosocomial Pathogens?

Nosocomial infections typically affect patients who are immunocompromised because of age, underlying diseases, or medical or surgical treatments. Aging of our population and increasingly aggressive medical and therapeutic interventions, including implanted foreign bodies, organ transplantations, and xenotransplantations, have created a cohort of particularly vulnerable persons. As a result, the highest infection rates are in intensive care unit (ICU) patients. Nosocomial infection rates in adult and pediatric ICUs are approximately three times higher than elsewhere in hospitals. The sites of infection and the pathogens involved are directly related to treatment in ICUs. In these areas, patients with invasive vascular catheters and monitoring devices have more bloodstream infections due to coagulase-negative staphylococci. In fact, most cases of occult bacteremia in ICU patients are probably due to vascular access-related infections. Fungal urinary tract infections have also increased in ICU patients, presumably because of extensive exposure to broad-spectrum antibiotics. In the National Nosocomial Infections Surveillance system, *Candida* spp. are the main cause of nosocomial urinary infections in ICUs (9).

### Why Are Nosocomial Infections Emerging Now?

Three major forces are involved in nosocomial infections. The first is antimicrobial use in hospitals and long-term care facilities. The increased concern about gram-negative bacilli infections in the 1970s to 1980s led to increased use of cephalosporin antibiotics. As gram-negative bacilli became resistant to earlier generations of cephalosporin antibiotics, newer generations were developed. Widespread use of cephalosporin antibiotics is often cited as a cause of the emergence of enterococci as nosocomial pathogens. About the same time, MRSA, perhaps also in response to extensive use of cephalosporin antibiotics, became a major nosocomial threat. Widespread empiric use of vancomycin, as a response to concerns about MRSA and for treatment of vascular catheter–associated infection by resistant coagulase-negative staphylococci, is the major initial selective pressure for VRE. Use of antimicrobial drugs in long-term care facilities and transfer of patients between these facilities and hospitals have created a large reservoir of resistant strains in nursing homes.

Second, many hospital personnel fail to follow basic infection control, such as hand washing between patient contacts. In ICUs, asepsis is often overlooked in the rush of crisis care (10).

Third, patients in hospitals are increasingly immunocompromised. The shift of surgical care to outpatient centers leaves the sickest patients in hospitals, which are becoming more like large ICUs (11). This shift has led to the greater prevalence of vascular access–associated bloodstream infections and ventilator-associated pneumonias.

Other precipitating factors also can be anticipated in hospitals. Transplantation is a double-edged sword because of the combined effects of immunosuppression of transplant patients and of infectious diseases that come with some transplanted organs. The blood supply will continue to be a source of emerging infectious diseases. Moreover, as hospitals age, infrastructure repairs and renovations will create risks of airborne fungal diseases caused by dust and spores released during demolition and construction. Infections due to other pathogens, such as Legionella, may also result from such disruptions.

### How Can We Prevent and Control Emerging Nosocomial Infections?

Infection control can be very cost-effective. Approximately one third of nosocomial infections are preventable. To meet and exceed this level of prevention, we need to pursue several strategies simultaneously (12). First, we need to continue to improve national surveillance of nosocomial infections so that we have more representative data. We must assess the sensitivity and specificity of our surveillance and of our case definitions, particularly for difficult-to-diagnose infections like ventilator-associated pneumonia. We also need to develop systems for surveillance of "nosocomial" infections that occur out of the hospital, where much health care is now given.

Second, we need to ensure that surveillance uses are valid. The Joint Commission on Accreditation of Healthcare Organization's ORYX initiative for monitoring health-care processes and outcomes will lead to core indicators and sentinel event monitoring. This initiative will be followed by increased outpatient surveillance, which ultimately may lead to systemwide real-time surveillance and reporting. Because we want to use nosocomial infection rates as a core indicator of quality of care, we need to improve our ability to "risk adjust" infection rates so we know that our interprovider and interhospital comparisons are valid. Risk stratification will ultimately depend on organic-based computer systems that will mimic biologic events.

Third, many of our successes in controlling nosocomial infections have come from improving the design of invasive devices. This is particularly important given the marked increase in frequency of vascular access–associated bloodstream infections, particularly in ICU patients. Given the choice of changing human behavior (e.g., improving aseptic technique) or designing a better device, the device will always be more successful. Of particular importance is the development of noninvasive monitoring devices and minimally invasive surgical techniques that avoid the high risk associated with bypassing normal host defense barriers (e.g., the skin and mucous membranes).

Fourth, forestalling the postantibiotic era will require aggressive antibiotic control programs (13); these may become mandated for hospitals that receive federal reimbursements, as happened in the past with infection control programs. Risks for antibiotic-resistant strains also may be reduced in the future by controlling colonization through use of immunization or competing flora.

Fifth, antimicrobial resistance problems and the advent of xenotransplantation emphasize the importance of newer microbiologic methods. For investigation of outbreaks of multidrug-resistant pathogens, pulsed-field gel electrophoresis has become a routine epidemiologic tool (14). Molecular epidemiologic analysis also may help us better understand the factors that lead to the emergence of resistant strains. For diagnosis of syndromes caused by unusual pathogens, representational difference analysis and speciation by use of the pathogen's phylogenetic r-RNA "clock" may become routine.

Sixth, control of tuberculosis (TB) in hospitals is an excellent example of the successful collaboration of the infection control community, CDC, and regulatory agencies. But we can anticipate that the Occupational Safety and Health Administration may have many new employee health issues—beyond TB and bloodborne pathogens—to evaluate in hospitals, such as health problems related to exposure to magnetic fields, to new polymers, and to medications that contaminate the environment. Problems of mental stress due to unrelenting exposure to pagers, faxes, e-mail, holograms, and telephonic implanted communicators will require special attention.

### Conclusion

Several enduring truths characterize the field of infection control. Hospitals will become more like ICUs, and more routine care will be delivered on an outpatient basis. Given the choice of improving technology or improving human behavior, technology is the better choice. All infection control measures will need to continue to pass the test of the "four Ps" (15): Are the recommendations Plausible biologically (e.g., is it likely to work)? Are they Practical (e.g., are they affordable)? Are they Politically acceptable (e.g., will the administration agree)? And, will Personnel follow them (e.g., can they and will they)?

The major advances in overall control of infectious diseases have resulted from immunization and improved hygiene, particularly hand washing. We must work with hospital personnel on better implementation of existing infection control technologies so that we will not need to rely solely on technologic advances.

Dr. Weinstein is chair, Division of Infectious Diseases, Cook County Hospital; director of Infectious Diseases Services for the Cook County Bureau of Health Services; and professor of Medicine, Rush Medical College. He also oversees the CORE Center for the Prevention, Care and Research of Infectious Disease and directs the Cook County Hospital component of the Rush/Cook County Infectious Disease Fellowship Program. His areas of research include nosocomial infections (particularly the epidemiology and control of antimicrobial resistance and infections in intensive care units) and health-care outcomes for patients with HIV/AIDS.

### References

1. Haley RW, Culver DH, White J, Morgan WM, Amber TG, Mann VP, et al. The efficacy of infection surveillance and control programs in preventing nosocomial infections in US hospitals. Am J Epidemiol 1985;121:182-205.
2. Haley RW, Culver DH, White JW, Morgan WM, Emori TG. The nationwide nosocomial infection rate: a new need for vital statistics. Am J Epidemiol 1985;121:159-67.
3. New York Times 1998 Mar 12; Sect. A12.
4. Centers for Disease Control and Prevention, Hospital Infections Program. National Nosocomial Infections Surveillance (NNIS) report, data summary from October 1986-April 1996, issued May 1996: A report from the NNIS System. Am J Infect Control 1996;24:380-8.
5. Slaughter S, Hayden MK, Nathan C, Hu TC, Rice T, Van Voorhis J, et al. A comparison of the effect of universal use of gloves and gowns with that of glove use alone on acquisition of vancomycin-resistant enterococci in a medical intensive care unit. Ann Intern Med 1996;125:448-56.
6. Bonten MJM, Hayden MK, Nathan C, Van Voorhis J, Matushek M, Slaughter S, et al. Epidemiology of colonisation of patients and environment with vancomycin-resistant enterococci. Lancet 1996;348:1615-9.
7. Hiramatsu K, Aritaka N, Hanaki H, Kawasaki S, Hosoda Y, Hori S, et al. Dissemination in Japanese hospitals of strains of *Staphylococcus aureus* heterogeneously resistant to vancomycin. Lancet 1997;350:1670-3.
8. Webster RG. Influenza: an emerging disease. Emerg Infect Dis 1998;4(3).
9. Fridkin SK, Welbel SF, Weinstein RA. Magnitude and prevention of nosocomial infections in the intensive care unit. Infect Dis Clin North Am 1997;11:479-96.
10. Weinstein RA. Epidemiology and control of nosocomial infections in adult intensive care units. Am J Med 1991;91:179-84.

11. Archibald L, Phillips L, Monnet D, McGowan JE, Tenover F, Gaynes R. Antimicrobial resistance in isolates from inpatients and outpatients in the United States: increasing importance of the intensive care unit. Clin Infect Dis 1997;24:211-5.
12. Scheckler WE, Brimhall D, Buck AS, Farr BM, Friedman C, Garibaldi RA, et al. Requirements for infrastructure and essential activities of infection control and epidemiology in hospitals: a consensus panel report. Infect Control Hosp Epidemiol 1998;19:114-24.
13. Goldmann DA, Weinstein RA, Wenzel RP, Tablan OC, Duma RJ, Gaynes RP, et al. Strategies to prevent and control the emergence and spread of antimicrobial-resistant microorganisms in hospitals. A challenge to hospital leadership. JAMA 1996;275:234-40.
14. Tenover FC, Arbeit RD, Goering RV. How to select and interpret molecular strain typing methods for epidemiological studies of bacterial infections: a review for healthcare epidemiologists. Infect Control Hosp Epidemiol 1997;18:426-39.
15. Weinstein RA. SHEA consensus panel report: a smooth takeoff. Infect Control Hosp Epidemiol 1998;19:91-3.

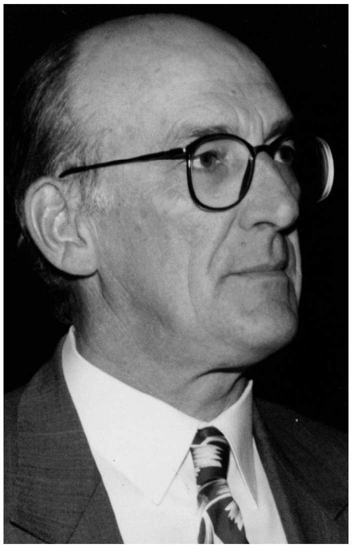

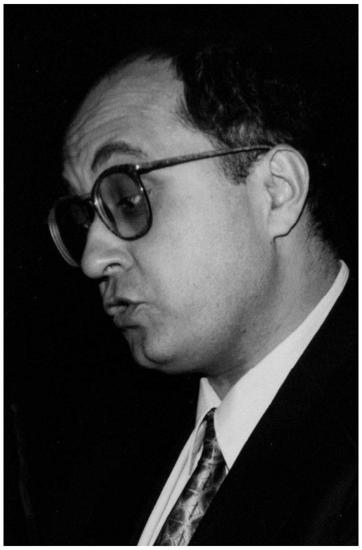

Sir John Pattison (L)
Medical School of University College, London
Sherif Zaki
Centers for Disease Control and Prevention, Atlanta, Georgia, USA
